# Weaknesses in the Reporting of Cross-sectional Studies in Accordance with the STROBE Report (The Case of Congenital Anomaly among Infants in Iran): A Review Article

**Published:** 2018-12

**Authors:** Morvarid IRANI, Maryam HASSANZADEH BASHTIAN, Talat KHADIVZADEH, Hosein EBRAHIMIPOUR, Seyyed Mohsen ASGHARI NEKAH

**Affiliations:** 1. Student Research Committee, Dept. of Midwifery, School of Nursing and Midwifery, Mashhad University of Medical Sciences, Mashhad, Iran; 2. Nursing and Midwifery Care Research Center, Dept. of Midwifery, School of Nursing and Midwifery, Mashhad University of Medical Sciences, Mashhad, Iran; 3. Social Determinants of Health Research Center, Dept. of Health Economic and Management Sciences, School of Health, Mashhad University of Medical Sciences, Mashhad, Iran; 4. Dept. of Educational and Counseling Psychology, School of Educational and Sciences Psychology, Ferdowsi University of Mashhad, Mashhad, Iran

**Keywords:** Iran, Epidemiology, Prevalence, Congenital anomaly, STROBE

## Abstract

**Background:**

The inadequate reporting of cross-sectional studies, as in the case of the prevalence of Congenital Anomaly, could cause challenges in the synthesis of new evidence and make possible mistakes in the creation of public policies. This study was conducted to critically appraise the quality of the articles involving congenital anomaly prevalence in Iranian infants with the STROBE recommendations.

**Methods:**

We performed a thorough literature search using the words “congenital anomaly” “birth defect” and “Iran” in MEDLINE/PubMed, Scopus, SID, Elmnet, Magiran, IranDoc, Iranmedex, and Google Scholar until Aug 2017. In this critical appraisal we focused on cross-sectional studies that reported the prevalence of congenital anomaly in Iranian infants. Data were analyzed using the STROBE score per item and recommendation.

**Results:**

The results of 17 selected articles on Congenital Anomaly prevalence showed that the overall accordance of the cross-sectional study reports with STROBE recommendations was about 63%. All articles met the recommendations associated with the report of the study’s rationale, objectives, setting, key results and provision of summary measures. Methods and results were the weakest part of the articles, in which recommendations associated with the participant flowchart and missing data analysis were not reported. The recommendations with the lowest scores were those related to the sensitivity analysis (6%, n=1/17), bias (6%, n=1/17), and funding (41%, n=7/17).

**Conclusion:**

Cross-sectional studies about the prevalence of congenital anomaly in Iranian infants have an insufficient reporting on the methods and results parts. We recognized a clear need to increase the quality of such studies.

## Introduction

The insufficient reporting of medical research is a long-lasting and potentially serious universal problem that is not evident to many researchers (1). All scientific study must be fully and precisely reported, letting a proper understanding of their methodology, findings, and repetition of the same if needed (2, 3). However, some of the reports are far from those standards (2). Therefore, many instructions that seek to standardize and progress the reporting quality of different kinds of study were established in the past few years (4).

Strengthening the Reporting of Observational studies in Epidemiology (STROBE) is an instruction whose recommendations have been presented for the purpose of sufficiently report observational studies (cohort, case-control, and cross-sectional studies) (3,5). STROBE recommendations evaluate the quality of reporting, but not the methodological quality (6). Furthermore, an insufficient reporting of cross-sectional studies could make possible mistakes in the synthesis and acceptance of new evidence and cause inaccuracies in the validation and the creation of public policies (2), particularly in areas with inadequate sources like Iran. For example, the prevalence of Congenital Anomaly (CA) is relevant to public health subjects because it had been related to the main causes of disability and mortality among children in developing countries (7, 8).

CA is the main cause of infant mortality; therefore, 21% of mortality during infancy results from these anomalies (9). This type of anomalies is the fifth leading reason for diminished natural life before the age of 65 and is one of the main causes of disabilities (10, 11). Costs of hospitalization and treatment events for these children carry out a large extra burden on the health system and their families. On the other hand, the diversity of the methods used for diagnosing anomalies and different characteristics of studied populations (live or dead babies) (12–14) related to the inadequate reporting in cross-sectional studies; create mistake when interpreting the actual scope of the problem. For that reason, this study aimed to appraise the reporting quality of cross-sectional studies on the subject of the prevalence of CA in Iranian infants, by the STROBE recommendations as an objective tool.

## Materials and Methods

This descriptive study was done in two stages. First, a systematic literature search was carried out to recognize the articles to be taken in the study. Then, the quality of the studies was evaluated with STROBE. This study keeps the recommendations of the PRISMA statement for its reporting (15).

### Data sources and search strategy

MEDLINE/PubMed and Scopus were searched for published cross-sectional studies. Search keywords were “Congenital anomaly”, “birth defect”, “prevalence”, and “Iran.” Persian databases (SID, Iranmedex, MagIran, Elmnet, and Iran Doc) and Google Scholar were also searched using equivalent keywords from 2007 until Dec 2017. In addition, reference section of relevant studies was manually checked to identify further studies missed by the electronic search. Authors were contacted for additional missing data. This study was performed independently and simultaneously by three researchers (MI, MHB, and TKH) and a list of found objects was made. Then, search results were assessed, and no differences in the outcome were obtained between the three authors.

### Study selection

Full-text articles were assessed by three researchers (MI, MHB, and TKH) and those who met the inclusion criteria were selected. Moreover, a secondary search through the bibliographic references of the chosen articles was done, and duplicates were removed. The inclusion criteria included descriptive and cross-sectional studies on the prevalence of congenital the anomalies among infants in Iran; studies were in English and Persian. The exclusion criteria included those studies that mentioned before publishing STROBE’s statement (Since 2007, STROBE’s statement has been published); studies that investigated the prevalence of congenital anomalies in the animal; qualitative studies, studies presented in conferences; and interventional studies. Short communications, editorials or reviews were excluded.

### Instrument

We worked the STROBE items for assessing the quality of the reports. STROBE offers 32 items for the suitable reporting of observational studies. These suggestions express the appropriate method of reporting the title, abstract, introduction, methods, results, discussion, and financing section (5). Based on the language of each reporting, we used the cross-sectional studies suggested-version, available in Persian or English (3). For this study, we operated 28 of the 32 recommendations from cross-sectional studies. We considered as not-applicable the items 16b (continuous variables were not categorized), 16c (the objectives of the studies were not to calculate the report of relative or absolute risk), 12d (sampling strategy was single-stage) and 13b (participation in study does not have multistage stage).

### Data extraction

Two methods were used to extract data. The first included information on the general characteristics of each article: first author, name of the study from which the data came from, publication year and language, city, study period, sample size and type of population. The second method is a list of 28 of 32 STROBE items. Three investigators (MI, MHB, and TKH) appraised the full-text of the articles, and the data was extracted. Each researcher considered whether the reports identified met or not the STROBE items. Lastly, the corresponding author of each included article was emailed. In each email, we gave the purpose of the study and the items completed by the article, according to our analysis based on STROBE. This step was performed with the purpose of clarifying potential contrasts with our appraisal. The responses of each author were evaluated based on the methodology expressed above and revisions to our analysis were done as accurate. If no response, a reminder email has been sent 8 d after the first one. We waited for 15 d for the authors to reply, and then our analysis was performed as the ending result.

### Analysis

Two kinds of scores were described including score per article and per item. The score per article was described as the number of the STROBE items sufficiently reported, divided by the total of items applicable per article and stated as a percentage. The score per item was described as the number of articles that met each STROBE item, divided by the total of articles for which the item was applicable and stated as a percentage.

## Results

**Fig. 1:**
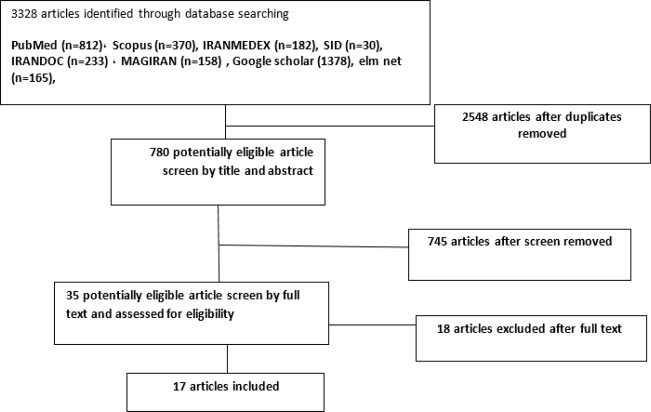
Article selection flow chart

We obtained 3328 articles within the database search. From these articles, 2548 were excluded for being duplicates and 754 were excluded for screen by title and abstract and the remaining 35 were checked in full-text. Of these, 18 were rejected because they did not meet the selection criteria, as a result, 17 articles were obtained for extracting information (16–32) (Fig. 1).

### General structures of the reports

Table 1 summarizes the main structures of the 17 included articles. Most Publication year of articles related to 2013 (23.5%) and 2014 (18%). Of the total, 5 articles (30%, n=4/17) (16, 19, 23, 27,31) were published in in MEDLINE/PubMed, 4 articles (23.5%, n=4/17) (17, 21, 25, 32) in Scopus, one article (6%, n=1/17) (30) in ISI databases and the others (35%, n=7/17) in Persian databases (SID, Elm net, Magiran, Irandoc, Iranmedex) (18, 20, 22, 24, 26, 28, 29). Only one article (6%, n=1/17) (20) had used a statistician in the Author List. Mean±SD of the period of reviewing articles was 6±5.3 months and the maximum period was 26 months (24), and the minimum was 1.5 months (18). These studies were done between 2000 and 2014 and included 189113 participants from different Iranian cities, involving urban and rural population. According to the publication language, six articles (35.3%) were published in English and 11 articles (64.7%) in Persian.

**Table 1:** Main structures of the articles about the prevalence of congenital anomaly in Iran

***Authors name***	***Publication year***	***Publication language***	***Study period***	***City***	***Population***	***age***	***Sample Size***
Mashhadi et al (16)	2014	English	2004–2012	Tabriz	rural	Children(live birth) under eight years of age	22500
Mohammadzadeh et al (17)	2013	Persian	2007–2008	Babol	Urban, rural	Newborn(live birth	1684
Khoshhal-Rahdar et al (18)	2014	Persian	2013–2014	Dezful	Urban, rural	Newborn(live birth	4235
Karbasi et al (19)	2009	English	October 2003 to June 2004	Babol	Urban, rural	Newborn(live birth and stillbirth)	4800
Akbarzadeh et al (20)	2008	Persian	2006–2007	Sabzevar	Urban, rural	Newborn(live birth	7786
Alijahan et al (21)	2013	Persian	2010–2011	Ardabil	Urban, rural	Newborn(live birth	6868
Hosseini et al (22)	2014	Persian	2012	Sistan	Urban, rural	Newborn(live birth	1800
Ahmadzadeh et al (23)	2008	English	2003–2006	Ahwaz	Urban, rural	Newborn(live birth	4660
Masoodpoor et al (24)	2013	Persian	2007–2008	Rafsanjan	Urban, rural	Newborn(live birth	6089
Tayebi et al (25)	2016	English	2008	Yazd	Urban, rural	Newborn	1195
Kavianyn et al (26)	2016	Persian	2008–2011	Golestan	Urban, rural	Newborn(live birth	92420
Jalali et al (27)	2011	Persian	2011	Rasht	Urban, rural	Newborn(live birth	1824
Sarrafan et al (28)	2011	Persian	2006–2007	Ahvaz	Urban, rural	Newborn(live birth	5087
Amini Nasab et al (29)	2014	Persian	2007–2011	Birjand	Urban	Newborn(live birth	22076
Dastgiri et al (30)	2007	English	2000–2004	Tabriz	Urban, rural	Newborn	1574
Rostamizadeh et al (31)	2017	English	2002–2003 2012–2013	Azarshahr	Urban, rural	Newborn	4515
Gheshmi et al (32)	2012	Persian	2007	Bandar Abbas	Urban, rural	Newborn(live birth	7007

### Reporting Quality based on the STROBE items

Eleven (56%) out of the 17 corresponding authors answered, mentioning and supporting if they approved (5/11) or opposed (6/11) with our analysis. Most of the conflicts were found in the items associated with the statistical analysis (the analysis of sensitivity, subgroups and missing data). According to these conflicts, each item was assessed again, and answers were emailed with the respective revisions. Table 2 shows the number of articles that met each STROBE item. The results of 17 selected articles on the prevalence of CA showed that the overall accordance of the cross-sectional study reports with STROBE items was about 63%. The highest score of those articles was 85% (21) and the least score was 42% (28). The most common weakness in the reporting quality was related to methodology and results estimated to be about 54% and 52%, respectively. The items that were fully met were those related to the reporting of the reasons and rationale for the investigation (item 2), to the reporting of the objectives (item 3), to the reporting of the setting (item 5), to provide summary measures (item 15) and summary key results (item 18). On the other hand, the items not reported were those associated with explaining the analysis of the missing data (item 12c), to consider the use of a flowchart for the participants (item 13c) and to indicate the number of participants with missing data for each variable (item 14b). The items with the lowest scores were those associated with the description of the sensitivity analysis (item 12e; 1/13 [6%]), to specify the steps taken to identify possible sources of bias (item 9; 1/17 [6%]) and to give the sources of funding (item 22; 7/17 [41%]).

**Table 2:** Number of articles that fulfill each item of the STROBE Statement

***Section***	***Subsection***	***Code***	***item***	***Fulfill each STROBEitem n%***
		1a	Indicate the study’s design with a commonly used term in the title or the abstract	14 (82)
**Title and abstract**	Title and abstract	1b	Provide in the abstract an informative and balanced summary of what was found	16(94)
	Background/rationale	2	Explain the scientific background and rationale for the investigation being reported	17 (100)
**Introduction**	Objectives	3	State-specific objectives, including any prespecified hypotheses	17 (100)
	Study design	4	Present key elements of study design early in the paper	13(76)
	Setting	5	Describe the setting, locations, and relevant dates, including periods of recruitment, exposure, follow-up, and data collection.	17 (100)
	Participants	6	Cross-sectional study: give the eligibility criteria and the sources and methods of selection of participants.	15(85)
	Variables	7	Clearly define all outcomes, exposures, predictors, potential confounders, and effect modifiers. Give diagnostic criteria, if applicable.	15(88)
**Methods**	Data sources/ measurement	8	For each variable of interest, give sources of data and details of methods of assessment (measurement). Describe comparability of assessment methods if there is more than one group.	16(94)
	Bias	9	Describe any efforts to address potential sources of bias.	1(6)
	Study size	10	Explain how the study size was arrived at	13(76)
	Quantitative variables	11	Explain how quantitative variables were handled in the analyses. If applicable, describe which groupings were chosen and why.	11(56)
	Statistical methods	12a	Describe all statistical methods, including those used to control for confounding.	10(60)
		12b	Describe any methods used to examine subgroups and interactions.	1(6)
		12c	Explain how missing data was addressed.	0(0)
		12d	Cross-sectional study: If applicable, describe analytical methods taking account of sampling strategy	NA
		12e	Describe any sensitivity analyses.	1(6)
	Participants	13a	Report numbers of individuals at each stage of study—e.g. numbers potentially eligible, examined for eligibility, confirmed eligible, included in the study, completing follow-up, and analyzed	16(94)
		13b	Give reasons for non-participation at each stage.	NA
**Results**		13c	Consider use of a flow diagram.	0(0)
	Descriptive data	14a	Give characteristics of study participants (e.g. Demographic, clinical, social) and information on exposures and potential confounders.	15(85)
		14b	Indicate a number of participants with missing data for each variable of interest.	0(0)
	Outcome data	15	Cross-sectional study: report numbers of outcome events or summary measures.	17(100)
	Main results	16a	Give unadjusted estimates and, if applicable, confounder-adjusted estimates and their precision (e.g. 95% confidence interval). Make clear which confounders were adjusted for and why they were included.	16(94)
		16b	Report category boundaries when continuous variables were categorized.	NA
		16c	If relevant, consider translating estimates of relative risk into absolute risk for a meaningful period.	NA
	Other Analyses	17	Report other analyses were done – e.g. Analyses of subgroups and interactions, and sensitivity analyses.	15(85)
	Key results	18	Summarize key results with reference to study objectives.	17 (100)
**Discussion**	Limitations	19	Discuss limitations of the study, taking into account sources of potential bias or imprecision. Discuss both direction and magnitude of any potential bias.	9(53)
	Interpretation	20	Give a cautious overall interpretation of results considering objectives, limitations, multiplicity of analyses, results from similar studies, and other relevant evidence.	15(85)
	Generalizability	21	Discuss the generalizability (external validity) of the study results.	8(46)
**Other information**	Funding	22	Give the sources of funding and the role of the funders for the present study and, if applicable, for the original study on which present article is based.	7(41)

## Discussion

The results of this study indicated that the performance of the STROBE items for the reporting of cross-sectional studies on the prevalence of CA was inadequate, the median STROBE score being 63%. Reporting rates were lowest for the methodology and results. These insufficiencies are mainly important for methodologically properly organized studies and correctly analyzed. For that purpose, every precise report, to be reliable, need to afford a strong, complete and obvious showing of what was designed, done and found, that simplify the sufficient understanding and publication of their results (3). Of the 17 articles including 189113 participants, all of them indicated limitations when reporting the methodology associated with the statistical analysis, involving sensitivity analysis, missing data, and sources of bias. Furthermore, the report of the results was not perfect about the explanation of the participant’s flow and participants with missing data for each variable.

The some studies evaluating the quality of observational study reporting, with the STROBE statement as a reference, identified a number of deficiencies harmonious with our results, involving marked insufficiencies in reporting the methodology and results (33–38), such as inadequate reporting in the management of missing data (35,36,39,40) and sources of bias (35,40). Preparing an ideal study report is the main responsibility of the authors, but several methods (editorial board and policies, external reviewers) play an important role in the publication process and also intention to a suitable report (41). To make this objective, the medical journal must follow reporting guidelines such as recommendations as an editorial policy; besides, reviewers and editors must be trained to its right usage (2). In general, the quality of reporting of cross-sectional studies could be enhanced if journals present an active policy of compliance with reporting recommendations such as STROBE (42, 43).

The academic reports make a greater purpose besides the production of new knowledge. Specifically, epidemiological researches have diverse attention, usages, and implications. For a more technical spectator, studies should report detailed estimates of the burden of the diseases that let ranking of public policies. Contrariwise, in case of more general spectators, they should offer a consistent implication about a particular condition. On both stages, technical and general spectators, we found that the articles analyzed about CA in Iran have significant restrictions in its report that reduce the suitable application of their findings.

The limitations of our study are that some of our findings might have been different if they were evaluated by other investigators; however, to prevent subjective decisions, each corresponding author was emailed to confirm our analysis, obtaining a rate response (56%). Additionally, we operated a global score for every article to provide a measure of whole reporting. In selecting this system, we do not suggest that all recommendations are of equal significance. On the other hand, we decided to use these policy help readers have an overall view of the quality of the reports and adherence to the STROBE statement in future studies has the potential to improve study reporting, help the appraisal and analysis of CA by reviewers, readers and journal editors, and finally support the practice of evidence-based medicine.

## Conclusion

Cross-sectional studies about the prevalence of CA in Iranian infants have an insufficient reporting of important parts such as methods and results. This finding indication a strong need to enhance the reporting quality of such studies to make its role to sufficiently inform relevant subjects for the putting into practice of public health policies.

## Ethical considerations

Ethical issues (Including plagiarism, informed consent, misconduct, data fabrication and/or falsification, double publication and/or submission, redundancy, etc.) have been completely observed by the authors.

## References

[1] Lang TA Secic M (2006). How to report statistics in medicine: annotated guidelines for authors, editors, and reviewers. 2nd ed ACP Press, United State of America. New York, pp.: 202–6.

[2] Glasziou P Altman DG Bossuyt P (2014). Reducing waste from incomplete or unusable reports of biomedical research. Lancet, 383(9913):267–276. 24411647 10.1016/S0140-6736(13)62228-X

[3] Von Elm E Altman DG Egger M (2014). The Strengthening the Reporting of Observational Studies in Epidemiology (STROBE) Statement: guidelines for reporting observational studies. Int J Surg, 12(12):1495–9. 25046131 10.1016/j.ijsu.2014.07.013

[4] Mannocci A Saulle R Colamesta V (2015). What is the impact of reporting guidelines on Public Health journals in Europe? The case of STROBE, CONSORT and PRISMA. J Public Health (Oxf), 37(4): 737–40. 25538144 10.1093/pubmed/fdu108

[5] Vandenbroucke JP von Elm E Altman DG (2007). Strengthening the Reporting of Observational Studies in Epidemiology (STROBE): explanation and elaboration. Epidemiology, 18(6):805–35. 18049195 10.1097/EDE.0b013e3181577511

[6] Costa BR Cevallos M Altman DG (2011). Uses and misuses of the STROBE statement: bibliographic study. BMJ Open, 1(1): e000048. 10.1136/bmjopen-2010-000048 PMC3191404 22021739

[7] Shokohi M Kashani KH (2001). Prevalence and risk factors of congenital malformations in Hamadan. J Mazandaran Univ Med Sci, 12(35):42–5.

[8] Irani M Khadivzadeh T Nekah A Mohsen S Ebrahimipour H Tara F (2018). The prevalence of congenital anomalies in Iran: A Systematic Review and Meta-analysis. IJOGI, 21(Supple):29–41.

[9] Al-Sadoon I Hassan G Yacoub A (1999). Depleted Uranium and health of people in Basrah: Epidemiological evidence. Incidence and pattern of congenital anomalies among births in Basrah during the period 1990–1998. MJBU, 17:27–33.

[10] Garry VF Harkins ME Erickson LL (2002). Birth defects, season of conception, and sex of children born to pesticide applicators living in the Red River Valley of Minnesota, USA. Environ Health Perspect, 110 Suppl 3: 441–9. 12060842 10.1289/ehp.02110s3441 PMC1241196

[11] Farhud D Walizadeh GR Kamali MS (1986). Congenital malformations and genetic diseases in Iranian infants. Hum Genet, 74 (4): 382–5. 3793101 10.1007/BF00280490

[12] Farhud D (1997). Evidence for a New AD Syndrome: Report of a Large Iranian Sibship with Severe Multiple Synostosis. Iran J Public Health, 26(1–2): 39–44.

[13] Farhud D Hadavi V Sadighi H (2000). Epidemiology of neural tube defects in the world and Iran. Iran J Public Health, 29(1–4): 83–90.

[14] Afshar M Golalipour MJ Farhud D (2006). Epidemiologic aspects of neural tube defects in South East Iran. Neurosciences (Riyadh), 11(4): 289–92. 22266439

[15] Liberati A Altman DG Tetzlaff J (2009). The PRISMA statement for reporting systematic reviews and meta-analyses of studies that evaluate health care interventions: explanation and elaboration. BMJ, 339:b2700. 19622552 10.1136/bmj.b2700 PMC2714672

[16] Mashhadi Abdolahi H Kargar Maher MH Afsharnia F Dastgiri S (2014). Prevalence of congenital anomalies: a community-based study in the Northwest of Iran. ISRN Pediatr, 2014:920940. 24995131 10.1155/2014/920940 PMC4005020

[17] Mohammadzadeh I Sorkh H Alizadeh-Navaei R (2013). Prevalence of external congenital malformations in neonates born in Mehregan Hospital, North of Iran. Genetics In The 3rd Millennium, 11(1):2990–95.

[18] Khoshhal-Rahdar F Saadati H Mohammadian M (2014). The Prevalence of Congenital Malformationsin Dezful-2012. Genetics In The 3rd Millennium, 12(2):3622–31.

[19] Karbasi SA Golestan M Fallah R (2009). Prevalence of congenital malformations. Acta Med Iran, 47(2):149–53.

[20] Akbarzadeh R Rahnama F Hashemian M Akaberi A (2008). The incidence of apparent congenital anomalies in neonates in mobini maternity hospital in sabzevar, iran in 2005–6. Journal of Sabzevar University of Medical Sciences, 15 (4): 231–6.

[21] Alijahan R Mirzarahimi M Ahmadi-Hadi P Hazrati S (2013). Prevalence of Congenital Abnormalities and Its Related Risk Factors in Ardabil, Iran, 2011. Iranian Journal of Obstetrics, Gynecology and Infertility, 16 (54): 16–25.

[22] Hosseini S Nikravesh A Hashemi Z Rakhshi N (2014). Race of apparent abnormalities in neonates born in Amir-almomenin hospital of Sistan. J North Khorasan Univ Med Sci, 6(3):753–579.

[23] Ahmadzadeh A Zahad S Masoumeh A Azar A (2008). Congenital malformations among live births at Arvand Hospital, Ahwaz, Iran-A prospective study. Pak J Med Sci, 24(1):33–37.

[24] Masoodpoor N Arab-Baniasad F Jafari A (2013). Prevalence and pattern of congenital malformations in newborn in Rafsanjan, Iran (2007–08). J Gorgan Univ Med Sci, 15(3):114–7.

[25] Tayebi N Yazdani K Naghshin N (2010). The prevalence of congenital malformations and its correlation with consanguineous marriages. Oman Med J, 25(1):37–40. 22125696 10.5001/omj.2010.9 PMC3215379

[26] Kavianyn N Mirfazeli A Aryaie M (2016). Incidence of birth defects in Golestan province. J Gorgan Univ Med Sci, 17(4): 73–77.

[27] Jalali S Fakhraie S Afjaei S Kazemian M (2011). The incidence of obvious congenital abnormalities among the neonates born in rasht hospitals in 2011. J Kermanshah Univ Med Sci, 19 (2): 109–7.

[28] Sarrafan N Mahdi-nasab A Arastoo L (2011). Evaluation of prevalance of congenital upper- and lower extremity abnormalies in neonatal live births in Imam and Razi hospital of Ahvaz. Jundishapur Sci Med J, 10 (70): 13–19.

[29] Amini Nasab Z Aminshokravi F Moodi M (2014). Demographical condition of neonates with congenital abnormalities under Birjand city health centers during 2007–2012. J Birjand Univ Med Sci, 21 (1): 96–103.

[30] Dastgiri S Imani S Kalankesh L Barzegar M Heidarzadeh M (2007). Congenital anomalies in Iran: a cross-sectional study on 1574 cases in the North-West of country. Child Care Health Dev, 33 (3): 257–61. 17439438 10.1111/j.1365-2214.2006.00720.x

[31] Rostamizadeh L Bahavarnia SR Gholami R (2017). Alteration in incidence and pattern of congenital anomalies among newborns during one decade in Azarshahr, Northwest of Iran. IJER, 4(1):37–43.

[32] Gheshmi AN Nikuei P Khezri M (2012). The frequency of congenital anomalies in newborns in two maternity hospitals in Bandar Abbas: 2007–2008. Genet 3rd Millennium, 9 (4): 2554–9.

[33] Tapia JC Ruiz EF Ponce OJ Malaga G Miranda J (2015). Weaknesses in the reporting of cross-sectional studies according to the STROBE statement: the case of metabolic syndrome in adults from Peru. Colomb Med (Cali), 46(4):168–75. 26848197 PMC4732506

[34] Egger M Altman DG Vandenbroucke JP (2007), Commentary: strengthening the reporting of observational epidemiology the STROBE statement. Int J Epidemiol, 36(5): 948–50. 17911150 10.1093/ije/dym199

[35] Fung AE Palanki R Bakri SJ Depperschmidt E Gibson A (2009). Applying the CONSORT and STROBE statements to evaluate the reporting quality of neovascular age-related macular degeneration studies. Ophthalmology, 116(2): 286–96. 19091408 10.1016/j.ophtha.2008.09.014

[36] Muller M Egger M (2009). Strengthening the reporting of observational epidemiology (STROBE) in sexual health. Sex Transm Infect, 85(3): 162–4. 19478105 10.1136/sti.2007.028985

[37] Papathanasiou AA Zintzaras E (2010). Assessing the quality of reporting of observational studies in cancer. Ann Epidemiol, 20(1): 67–73. 20006277 10.1016/j.annepidem.2009.09.007

[38] Jeelani A Malik W Haq I Aleem S Mujtaba M Syed N (2014). Cross-sectional studies published in Indian journal of community medicine: evaluation of adherence to strengthening the reporting of observational studies in epidemiology statement. Ann Med Health Sci Res, 4(6): 875–8. 25506479 10.4103/2141-9248.144889 PMC4250984

[39] Langan S Schmitt J Coenraads P-J Svensson Å von Elm E Williams H (2010). The reporting of observational research studies in dermatology journals: a literature-based study. Arch Dermatol, 146(5):534–41. 20479302 10.1001/archdermatol.2010.87

[40] Poorolajal J Cheraghi Z Irani AD Rezaeian S (2011). Quality of cohort studies reporting post the Strengthening the Reporting of Observational Studies in Epidemiology (STROBE) statement. Epidemiol Health, 33: e2011005. 21716598 10.4178/epih/e2011005 PMC3110877

[41] Glujovsky D Villanueva E Reveiz L Murasaki R (2014). Adherencia a las guías de informe sobre investigaciones en revistas biomédicas en América Latina y el Caribe. Rev Panam Salud Publica, 36(4): 232–7. 25563148

[42] Pouwels KB Widyakusuma NN Groenwold RH Hak E (2016). Quality of reporting of confounding remained suboptimal after the STROBE guideline. J Clin Epidemiol, 69:217–24. 26327488 10.1016/j.jclinepi.2015.08.009

[43] Hopewell S Ravaud P Baron G Boutron I (2012). Effect of editors’ implementation of CONSORT guidelines on the reporting of abstracts in high impact medical journals: interrupted time series analysis. BMJ, 344: e4178. 22730543 10.1136/bmj.e4178 PMC3382226

